# Modelling the prebiotic origins of regulation and agency in evolving protocell ecologies

**DOI:** 10.1098/rstb.2024.0287

**Published:** 2025-10-02

**Authors:** Ben Shirt-Ediss, Arián Ferrero-Fernández, Daniele De Martino, Leonardo Bich, Alvaro Moreno, Kepa Ruiz-Mirazo

**Affiliations:** ^1^ DIPC (Donostia International Physics Center -- CSIC, UPV/EHU), Donostia - San Sebastián E20018, Spain; ^2^ Biofisika Institute (CSIC, UPV/EHU), 48940 Leioa, Spain; ^3^ Ikerbasque, Bilbao 48009, Spain; ^4^ Department of Philosophy, University of the Basque Country, Donostia - San Sebastián E20018, Spain

**Keywords:** prebiotic systems evolution, minimal metabolism, ecopoiesis, regulation, adaptive agency, CRMs (consumer–resource models)

## Abstract

How and why did natural systems develop the first mechanisms of regulation? How could they turn into adaptive agents in a minimal (though deeply meaningful) biological sense? A novel simulation platform, Araudia, has been implemented to address these tightly interrelated questions, in a prebiotic scenario where metabolically diverse protocells are allowed to modify their dynamic behaviour in response to changes in their boundary conditions (e.g. nutrient concentrations in the medium) and/or in the activity of other protocells, including cross-feeding relationships. On these lines, we extend ‘consumer–resource models’ to a stochastic evolutionary framework in which novelty appears *bottom-up* (i.e. from small changes at the individual protocell level), and a short-term memory may also come forth and spread in the population, with the aim to demonstrate that simple (pre-genetic) adaptive/learning processes can have relevant effects at somatic times (i.e. within the lifetime of single protocells). Our interest in exploring the interplay between metabolic-physiological aspects and ecological-evolutionary ones derives from the fact that this provides a complex causal domain in which *the actual* and *the possible* talk to each other and, as the results clearly indicate, regulatory and agent capacities become crucial for the survival of infra-biological systems and their subsequent transition towards full-fledged life.

This article is part of the theme issue ‘Origins of life: the possible and the actual’.

## 1. Introduction

Life is autonomy in open-ended evolution [1]. This requires genetically instructed and cellularly organized metabolisms, whose emergence from physics and chemistry remains a mystery. Our way of tackling this deep scientific riddle favours the early formation of proto-cellular systems, as was argued for and analysed thoroughly in previous work (see [2,3] and references therein). Here we move to a more advanced prebiotic scenario where those relatively simple protocells, which may already display some self-productive and self-reproductive capacities [4], should transform into increasingly elaborate and efficient metabolic systems. As explained in more detail below, this involves addressing the problem of how natural systems generate, for the first time, phenotypic diversity, along with mechanisms to regulate their dynamic behaviour, so as to respond adaptively to the multiple perturbations coming from a highly variable environment and, thus, also enable plasticity. The conditions to produce phenotypic diversity and strategies for regulation/plasticity have been well studied in evolutionary biology for decades [5,6]. However, they have been rarely explored in the field of origins of life, and less so in connection with the appearance of the first natural agents—protocells, we claim—whose interactive robustness had to increase if they were to undergo major prebiotic transitions and eventually become full-fledged living beings [7,8].

In this context, it is important to realize that proto-cellular systems must have faced a strong bottleneck [9]: the higher their complexity, the larger the space of possible dynamic trajectories/behaviours available to each of them. At earlier stages in the transition from the inert to the living, the main difficulty was probably dealing with the combinatorial explosion in chemical composition and reactivity associated to proto-metabolic networks [10]; nevertheless, once that is overcome and a subset of organic building blocks is selected to construct molecular and organizational complexity, the challenge is to tame what we shall here call ‘functional expansion’ processes. This is not an issue for models of origins of life based on the evolution of self-replicating polymers, since the functional/phenotypic space of those molecular entities stays rather limited. Regardless of the amount of possible monomer combinations that one can theoretically calculate, for a given polymer length (which soon becomes a really vast number), phenotypic space typically remains two-dimensional: ‘resistance to hydrolysis’ and ‘replication speed’ of the molecules involved [11,12]. However, when one conceives abiogenesis as a process of protocell development [8,13], the individuals of the evolving population consist in compartmentalized micro-reactors, supramolecular entities with a heterogeneous distribution of component parts, whose repertoire of dynamic behaviours (i.e. number of possible stationary states, asymptotic attractors or, simply, viable reaction trajectories/configurations) significantly expands as they transform into increasingly complex organizations. Dynamic systems theory strongly supports this idea, having demonstrated, over the years, that the dimensionality and nonlinearities present in the description of a system correlate with features like multi-stability, complex behaviour, unexpected bifurcations, etc. (see [14] for a review on the topic). Furthermore, being open metabolic systems that synthesize their own physical boundaries, protocells must engage, in parallel, in well controlled exchanges with a variable environment—if they are to maintain themselves, grow and eventually reproduce under non-equilibrium conditions.

Thus, the way we envision the process of material and organizational complexification towards minimal life involves populations of self-(re-)producing protocells in continuous interaction with their peers and their inert—but variable—surrounding milieu, as a result of which they develop novel ways of thriving, and of operating functionally and adaptively as individuals, even though the effort required is necessarily collective and transgenerational [9]. This implies modifying both the internal metabolic dynamics of each protocell, as well as the external (competitive and cooperative) relationships that they establish with other members of the population. Therefore, a large *space of possibilities* will be, in principle, accessible to each protocell, even though only a relatively small *subregion of actual trajectories* can be, in practice, probed. Random or unguided searches in such a complex causal domain are bound to fail, sooner or later, unless the environment remains relatively stable. But this is not realistic in any natural, prebiotic setting—and less so if each individual is surrounded by similar ones, undergoing chemical transformations and continuous change. Therefore, the chances for these systems to increase their levels of material and organizational complexity strongly depend on the implementation of mechanisms to: (i) search effectively in the space of possible dynamic states/functions available to them, taking into account the inputs and perturbation patterns they get from their environment; and (ii) record and transmit reliably those mechanisms to subsequent generations in the population.

The computational approach and simulation results presented below will focus on the first point (i), assuming that protocells, if they manage to grow, will reproduce without difficulties and transmit to their offspring their composition, properties, the variations they occasionally suffer, as well as what they ‘learn’ on the way. We are aware that this is a crude simplification, and (ii)—namely, the appearance of hereditary mechanisms—will also deserve attention in future research. Yet, our primary aim here was to understand how regulation, as a second-order control mechanism [15], could contribute to the dynamic robustness and phenotypic plasticity of metabolizing protocells when ‘the possible’ becomes much larger than ‘the actual’. Thus, in this work, the chemical details of the mechanisms enabling that transition will be deliberately abstracted away, to concentrate on the functional consequences for the systems involved. Our general assumption is simply that the uptake and processing of nutrients into by-products is carried out by ‘first-order constraints’, precursor catalysts/transporter molecules (see the model description below, and the electronic supplementary material, for more details), whose performance must be modulated by an additional set of constraints if protocells are to cope/survive in a rapidly varying world where they have too many options at reach. In that context, ‘second-order’ regulatory mechanisms come to the rescue, channelling system responses according to the patterns of change—or the challenges—that the protocells get from the environment. More specifically, our central interest here will be to investigate the emergence of dynamic behaviours that can be tuned within the lifespan of a single protocell (i.e. ontogenetic or somatic adaptation), even if the process of establishment of the underlying regulatory mechanism across the population, the ‘assimilation–sedimentation’ process itself, may cover much longer, evolutionary timescales (i.e. protocell phylogenies of variable length).

In brief, our approach to the problem consists in offering an evolutionary framework (supported by a new stochastic simulation platform, Araudia), where the complexity of the individuals in the population not only concerns their internal metabolic nature (as compartmentalized, chemical micro-reactors) but also is linked to the proto-ecological relationships that they set up (in particular, cross-feeding), which allow the co-existence of diverse protocell types within the same environment. For that purpose, our computational platform was built as an elaboration of microbial ‘consumer–resource models’ (CRMs) [16–18], which try to account for the high levels of species diversity observed in natural ecosystems, moving beyond the classical ‘competitive exclusion principle’ that applies in most evolutionary models (see the critical review [19] and references therein). Keeping metabolically and ecologically distinct species in the pool of evolving protocells is important for our origins-of-life research programme, since we also aim to explore the emergence and evolutionary development of ‘minimal autonomous agency’ [7,20,21] within the same prebiotic setting. Indeed, regulatory mechanisms that modulate, specifically, the *outward* behaviour of single protocells (including the interactive dynamics they engage into with their peers) must have constituted a basic requirement for the first adaptive forms of agency to ever unfold in nature and further evolve.

But one step at a time: let us introduce briefly first the main characteristics and assumptions of our computational tool, our stochastic CRM, where occasional changes in the values of the parameters associated to single protocells create ‘variants’ that drive evolution from the bottom-up. In addition to several metabolic constraints that make the model thermodynamically consistent, we include a plastic ‘short-term memory’ to channel system responses to environmental challenges by tuning the amounts of functional components present in each protocell—well, more precisely, their *rates* of change. Although we will remain agnostic about the chemical nature of such a memory mechanism, various sources of evidence (both theoretical and experimental: e.g. [22–25]) support the hypothesis that, even in the absence of molecular templates, or genetics, chemical reaction networks may implement learning/recording capacities. Anyhow, as we advanced above, our central goal here is to show that the presence of this second-order flexible constraint (main novelty of our approach with regard to previous CRMs) can lead to regulatory behaviours of the ‘lac-operon’ kind, as we will report later, in the Results—together with other findings that involve minimalist protocell ecologies. Finally, in the Discussion and outlook, we recapitulate the main outcomes of our simulation work, analyse their theoretical implications and suggest future lines of investigation that will expand beyond the rather elementary ‘proof-of-principle’ cases so far explored.

## 2. Computational methods and model description

**Figure 1.**
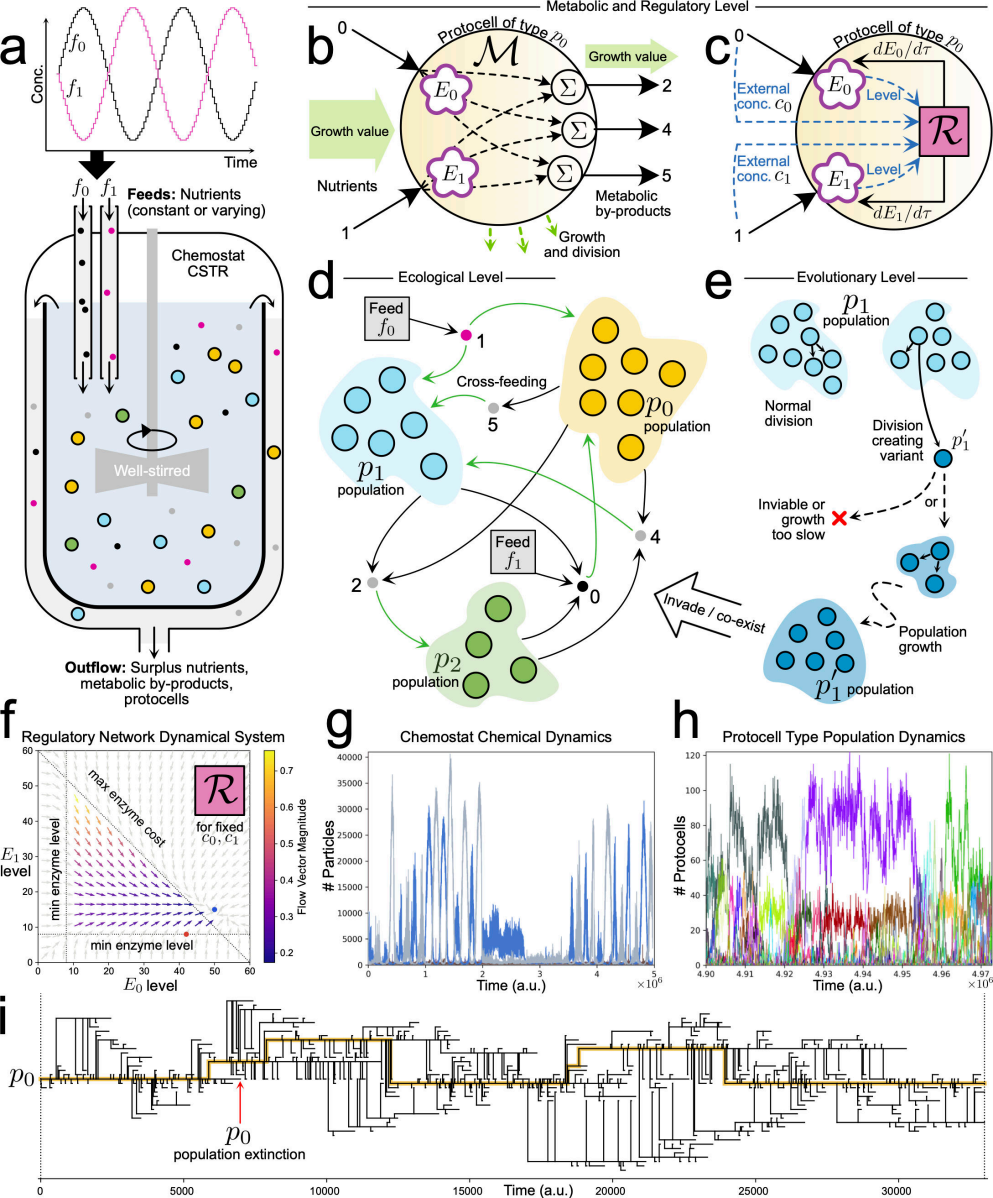
Modified consumer–resource model to study the emergence of minimal regulation strategies in an evolving ecology of protocells. (a) Protocell population dynamics take place in a well stirred constant-volume chemostat with varying nutrient inputs. Each protocell has (b) a metabolic network and (c) an (initially disabled) regulatory network (see text). (d) Populations of different protocell types can co-exist in the flow reactor by using strategies such as cross-feeding. (e) Furthermore, the current protocell ecology can be disrupted by evolutionary events whereby a variant protocell type is created and grows as a new population. Remaining panels show example dynamics from simulations: (f) enzyme-level dynamics of a regulatory network (see text); (g) nutrient and metabolic by-product chemical dynamics in the reactor; (h) zoom of protocell population dynamics in the reactor; (i) lineage tree showing the evolution of one protocell type in the initial condition, for a short time.

Our main effort for this contribution was to encompass, within a single simulation platform, several processes that happen at different spatial and temporal scales: from physicochemical transformations to protocell metabolisms to ecological networks; and from the on-going (somatic) behaviours of single protocells to much longer-term, transgenerational trajectories (i.e. evolving phylogenies). This necessarily implied taking some simplifying commitments, which have biased the research carried out, so it is very important to make them explicit. For starters, entities in our model are of three main types: *molecules* (chemicals: nutrients/by-products), *protocells* (larger micro-reactors that take up and process those chemicals through functional components or ‘enzymes’—which actually operate as a blend between catalyst and transporter) and *populations* (collections of identical protocells, each corresponding to a ‘sub-species’ biomass).^1^ As depicted in figure 1, the amounts of all of these entities change in time, stochastically, within a well stirred chemostat.

On top of an optimized Gillespie algorithm [26,27] implementing the basic reaction kinetics, our computational platform Araudia introduces the possibility of evolution by creating protocell ‘variants’ with modified parameter values. Such evolutionary changes are only allowed, with a certain probability, at division steps (which, in turn, only occur if there is sustained protocell growth) and will scale up to the population level (or not), becoming a significant piece of the biomass (or not), depending on how well the new protocell variant does in the chemical context it encounters at birth, and in the interactive dynamics it establishes with other (co-existing) protocell sub-species. If a protocell variant eventually gives rise to another variant that remains within the ‘protocell phylogeny trunk’ (yellow line in figure 1i), the former will be considered a *significant* variant, since its contribution to the protocell lineage is long term. Hence, a standard simulation begins with a number of protocells (typically, in the hundreds) of one or several species and, provided that they thrive, a ‘cloud of sub-species’ will be subsequently generated. All this involves quite rich and complex dynamics, reminiscent of the ‘quasi-species’ model [28], even if the underlying processes and assumptions here are completely different. Our individuals grow and divide into identical daughters, without errors, unless a variant appears, but they do not behave like ‘sequence replicators’: rather, they constitute compartmentalized chemical bioreactors (more precisely, the linear sum of the metabolic pathways that define each protocell—see figure 1b) evolving under variable external conditions and under the ‘selective pressure’ of being directly washed out, with a certain probability, from the tank (i.e. no explicit fitness function is employed, as was done in some related studies [29]—just a global ‘reactor flow speed’ or ‘dilution rate’).

For time periods over which (i) the protocell ecology in the reactor has a fixed number of protocell types (i.e. no new types are created, and no types go extinct) and (ii) the reactor feeds are constant in concentration, the dynamics of our model can be approximated by deterministic equations (2.1)–(2.6). These equations are derived from low-level stochastic events and follow the form of MacArthur’s CRM [30,31]. The concentration dynamics of chemical, $c_{i}$, inside the flow reactor is then described [29] by:


(2.1) $$\frac{dc_{i}}{d\tau }=\mu \left(f_{i}-c_{i}\right)-\gamma_{i}^{nutrient}+\gamma_{i}^{byproduct},$$

where μ is the dilution rate and $f_{i}$ is the concentration of chemical i in the reactor feed. Note that arbitrary units are used for time (τ) and concentration. Protocell populations in the reactor consume $c_{i}$ at the following overall rate:


(2.2) $$\gamma_{i}^{nutrient}=\sum_{\sigma \in N_{i}}p_{\sigma }\left(E_{\sigma i}\frac{c_{i}}{1+c_{i}}\right),$$

where $N_{i}$ is the set of all protocell types that import $c_{i}$ as a nutrient and $p_{\sigma }$ is the concentration of protocell type σ. Each protocell type imports a nutrient at a rate determined by the Monod equation: as external nutrient concentration increases, uptake rate saturates, with a maximum determined by the amount of ‘import-processing enzyme’, $E_{\sigma i}$, inside the protocell (where there are as many kinds of these enzymes as nutrients in the diet). Following the same assumption as in MacArthur’s consumer–resource formalism, all individuals within a protocell type population (or biomass) behave identically.

Protocell populations also excrete chemicals as metabolic by-products, which enables cross-feeding. The overall rate at which chemical $c_{i}$ is produced by all populations is given by:


(2.3) $$\gamma_{i}^{byproduct}=\sum_{\sigma \in B_{i}}\sum_{n\in N_{\sigma }}p_{\sigma }\left(E_{\sigma n}\frac{c_{n}}{1+c_{n}}\right)M_{ni}^{\sigma },$$

where $B_{i}$ is the set of all protocell types that excrete $c_{i}$ as a metabolic by-product and $N_{\sigma }$ is the set of chemicals that protocell type σ consumes as nutrients. Following [16], $M_{,}^{\sigma }$ encodes the metabolism of protocell type σ: it is a matrix of weights with $M_{ni}^{\sigma }$ denoting the weight between nutrient input n and by-product output i. As detailed in the electronic supplementary material, to ensure physical/thermodynamic consistency, we introduced constraints on the weight values in M such that spontaneous energy/mass creation in protocell ecologies is prohibited. In particular, we applied the rule that the overall rate of (nutrient) growth value flowing into any metabolism operating in the chemostat should be strictly higher than the overall rate of (by-product) growth value flowing out of it.

The concentration (population) dynamics of a protocell type is defined by the balance between growth rate and flow-reactor wash-out rate:


(2.4) $$\frac{dp_{\sigma }}{d\tau }=p_{\sigma }\left(g\lambda_{\sigma }-\mu \right),$$

where $\lambda_{\sigma }$ is the net growth value per unit time available for protocell type σ growth and g is a scale factor determining how quickly growth value derived from nutrients is converted into protocell biomass. The net growth value per unit time available for protocell growth is:


2.5 $$\lambda_{\sigma }=\left(\sum_{n\in N_{\sigma }}E_{\sigma n}\frac{c_{n}}{1+c_{n}}\Delta v_{\sigma n}\right)-R^{maintain}-\left(\sum_{n\in N_{\sigma }}E_{\sigma n}e_{n}\right),$$

which is derived from the growth value per unit time (i.e. the processed nutrients), minus (i) an overall fixed cost for self-maintenance, $R^{maintain}$, and (ii) a ‘resource cost’ related to the number of functional components (i.e. the ‘enzymes’) operating in each protocell. The metabolic matrix $M_{,}^{\sigma }$ determines parameter $\Delta v_{\sigma n}$, denoting how much useable growth value type σ can extract from nutrient n. It is important to highlight here that internal enzyme levels cannot be arbitrarily large and a maximum enzyme cost constraint is applied, which forces a trade-off in uptake-processing enzyme levels (similar to [32]; see also electronic supplementary material).

Thus, in the deterministic limit, our model corresponds to a standard microbial CRM (miCRM) that implements an ‘OR’ function for nutrients (i.e. protocells can feed on one or all nutrient inputs in their diet, and nutrient uptake rates are essentially independent of each other). However, all of this turns quite more complicated on the introduction of an additional, novel feature: a ‘memory function’ that operates as a flexible (second-order) constraint on the dynamics of each protocell type (i.e. affecting the nutrient preferences of that particular protocell type). Since this is a key aspect that makes our work distinct from previous CRMs, let us explain it a bit further.

Classical MacArthur CRMs have species with fixed nutrient preferences. However, with the aim to investigate the origin of regulatory mechanisms and their impact on the behaviour and adaptive capacities of proto-cellular systems, we searched for a plastic function that could correlate protocell states with changes/perturbations coming from the external medium—beyond their intrinsic potential to respond to those changes through random variations across evolutionary timescales. In other words, we wanted to include a possible source of ‘ontogenetic plasticity’ for the protocells (in addition to their inherent ‘phylogenetic adaptivity’—as self-reproducing, evolving systems). On these lines, instead of adding the possibility of modifying directly the number of functional components of a protocell, we thought that it was more interesting to modulate the *rate of change* of those components, so we used, for that purpose, a ‘neural ODE’, following [33]. The neural ODE (a network with variable weights, whose outputs are the values of the corresponding derivatives—see figure 1c,f), can be active or inactive, depending on the situation under analysis, but it is never explicitly trained: it is just ‘tinkered’ with through the actual evolutionary trajectories obtained in our simulations. So, even though we use a neural network, what we have implemented here is quite different to the practice of supervised machine learning: our neural ODE is just an abstract formulation of the high-order constraint (i.e. a correlation function) that can serve as a ‘memory’ for our protocells,^2^ if they manage to take advantage of it (which is not always the case).

Thus, the neural ODE in our simulation platform, although initially disabled (i.e. $dE_{\sigma i}/d\tau =0$), can be tinkered with through evolution to allow protocells to change their nutrient preferences in potentially very complex ways, based on the availability of external nutrients and its own internal enzyme state:


(2.6) $$\frac{1}{k_{\tau }}\frac{dE_{\sigma i}}{d\tau }=NN_{\sigma }^{i}\left(\left[E_{\sigma },c_{\sigma }\right]\right)-k_{\sigma i}E_{\sigma i}-\xi_{max}\left(E_{\sigma i}\right)+\xi_{min}\left(E_{\sigma i}\right),$$

where $NN_{\sigma }$ is a neural network function determining the production rate of import enzymes in protocell species σ, implemented as a multi-layered perceptron with a single hidden layer of five nodes. Parameter $k_{\tau }$ determines the response timescale. Protocell import/processing enzyme levels are also subject to (i) first-order decay controlled by constant $k_{\sigma i}$ and (ii) conditional forcing by functions $\xi_{min}$ and $\xi_{max}$ to ensure that protocells do not invalidate the maximum enzyme cost constraint mentioned above (see electronic supplementary material for a full description).^3^

The creation of new protocell types (speciation) is handled at the stochastic event level (each protocell division being a discrete event in our model). The mean rate at which a single protocell divides (with one of the two daughters harbouring random modifications) is one every $E_{div}$ divisions, for all protocell types. The modified daughter protocell is added to the reactor as a new protocell type in a single copy number, inheriting the characteristics of its parent type, but with small changes made to the weights in its metabolic network M and in its regulatory neural network NN (again, see electronic supplementary material for more details). Note that our formalism includes no explicit ‘genotype’, so these ‘mutations’ are carried out directly on high-level attributes M and NN: i.e. our protocells lack an underlying low-level representation of membrane biophysics and metabolic/regulatory networks that would, ultimately, give rise to such attributes. Finally, it is important to remark that speciation in our framework is naturally handled as part of the underlying event-by-event Gillespie stochastic simulation, ensuring a rigorous treatment of species birth and extinction—in contrast to other approaches within the MacArthur school (e.g. [35]), which have implemented it as a stochastic component able to add new species differential equations at each time step of the deterministic solver.

After this brief description of the model and methods used, we are ready to show, next, what our approximation to the problem has provided in terms of simulation results. All the data and plots for analysis reported below come from computer simulations carried out with Araudia running on an HPC cluster. Parameter settings can be found in the electronic supplementary material. In addition, the simulation code and all the results are available in the sources cited at the end of the article (‘Software availability’ section).

## 3. Results

The stochastic dynamics of three different minimal protocell ecologies were investigated, searching specifically for protocells that spontaneously evolve functional ‘lac-operon’-like regulatory networks as a way of enhancing their survival: i.e. regulatory networks that can quickly and flexibly switch the nutrient preference of a host protocell to match the most abundant nutrient in the environment. In this prebiotic context, the capacity for ‘nutrient preference switching’ can be seen as a rudimentary agent property (see §4), and one that can be explored, in any case, in the non-equilibrium and well stirred conditions assumed in our model.

### (a) General conditions for our ‘*in silico* chemostat’ experiments

Two nutrient chemicals were supplied to the flow reactor, either both at a constant level (at 200 concentration units each), or each varying as a sine wave, 180° out of phase with the other (sine waves between 40 and 200 concentration units). We tested two main cases: a −REG control case, where protocells were not allowed to develop regulatory networks, and a +REG case, where protocells started with disabled regulatory networks but had the potential to turn functional through ‘evolutionary tinkering’ of the neural ODE weights. In the −REG control case, the only way that protocells could adapt to changing nutrient abundance was by using phylogenetic adaptation (i.e. the birth of fitter, more suited variants) to modify the levels of their import/processing enzymes and metabolic network weights, over relatively long timescales. All results reported below measure enzyme dynamics across the entire evolutionary lineage of protocells, beginning with the protocell type(s) in the initial condition, as illustrated by the yellow line in figure 1i.

Our analysis typically focuses on internal enzyme dynamics, because they directly reflect the nutrient preferences of protocells. Although the evolving protocell system also has other interesting aspects, such as protocell population dynamics, reactor chemical dynamics, or the evolution of metabolic network weights, these are secondary to our aims here and are not explicitly reported. Finally, in all simulations described below, major evolutionary innovations were disabled and, therefore, each protocell variant imported the same nutrients and excreted the same metabolic by-products as its parent type. Nevertheless, loosely speaking, ‘ecologies’ of different protocell types or protocell ‘sub-species’, each with its corresponding biomass (see endnote^1^ ), are generated throughout our simulations.

### (b) Case study 1: ‘single-protocell species’

**Figure 2.**
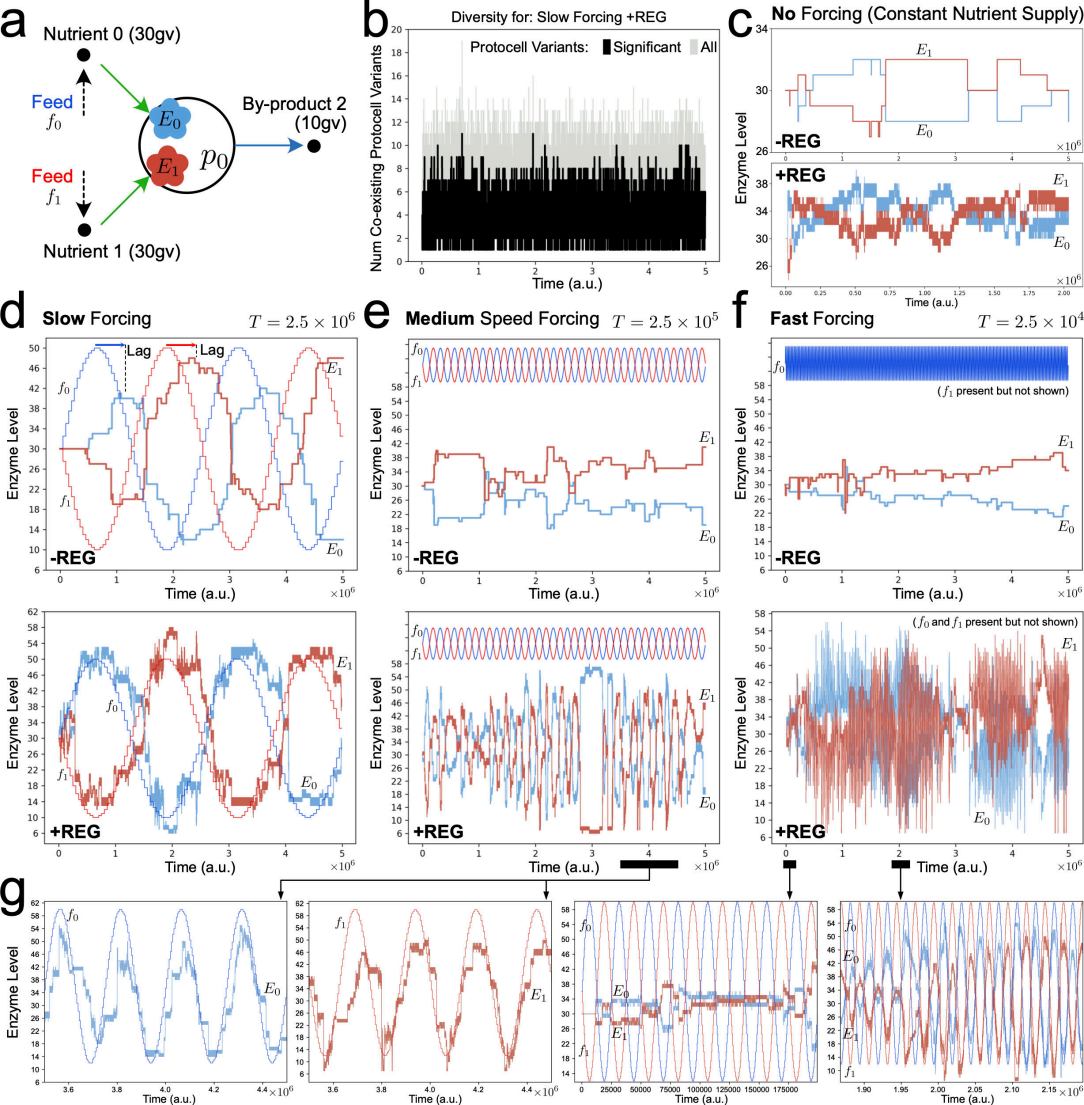
Dynamics of internal enzyme levels across an evolutionary lineage stemming from a single-protocell type, when protocells are deficient in regulatory networks (−REG) and when protocells have the potential to develop functional regulatory networks (+REG), under constant nutrient supply, and slow-, medium- and fast-varying nutrient forcing. (a) Reactor starts with a population (*n* = 100) of protocell variant $p_{0}$, which consumes nutrient 0 or nutrient 1, or both, to grow. (b) Many variants of $p_{0}$ arise and co-exist during the course of evolution. 'Significant' protocell variants are those that give rise to further variants. (c) Under constant nutrient input (reactor feeds *f*_0_ = *f*_1_ = 200 conc. units), enzyme levels across the evolutionary lineage remain equally split or diverge symmetrically in both −REG and +REG cases. (d–f) Enzyme dynamics across the evolutionary lineage when reactor feeds *f*_0_ and *f*_1_ now vary as sine waves, between 40 and 200 conc. units, 180° out of phase. When sinusoidal forcing is slow, −REG protocells can still slowly adapt via phylogenetic changes (d, upper graph), but faster nutrient forcing prohibits phylogenetic adaptation (e, f, upper graphs). Conversely, under faster nutrient forcing, +REG protocells evolve ‘lac-operon’-like dynamics, allocating (limited) internal enzyme levels in phase with the varying abundance of nutrients in the environment (e,f, lower graphs). (g) Zoom plots of regulatory network enzyme dynamics in response to nutrient forcing. Note that nutrient forcing sine waves *f*_0_ and *f*_1_ use a separate *y*-axis that is not shown.

The first and simplest scenario we investigated was the evolution of a population of a single-protocell species $p_{0}$ that could feed on either of the supplied nutrients 0 or 1, or both of them (figure 2a). Each of the supplied nutrients had equal growth value. Protocell type $p_{0}$ already possessed a passive ‘null strategy’ to survive in changing nutrient abundance conditions: it could import and grow from the nutrient present at the time, and simply pay the maintenance cost for the import enzyme associated with the non-present nutrient. However, evolutionary development of a regulatory network could enable a more sophisticated and efficient response: namely, dynamic allocation of the limited import enzyme budget toward the currently more abundant nutrient in the environment.

When the reactor feeds supplied constant nutrient levels (at 200 concentration units each), in both the −REG and +REG cases, the *E*0 and *E*1 enzyme levels both either evolve to hover around the midpoint of 30 units (figure 2c), or diverge symmetrically from the midpoint. This divergence can be rationalized by the fact that nutrients 0 and 1 had equal growth values, and under constant nutrient supply they could be imported almost interchangeably. As we advanced above, even in this simple case of a single-protocell species, in which the population is under constant nutrient supply, the initial biomass (approx. 100 identical protocells) systematically evolves to diverse pieces of biomass over time: that is, from a single-protocell type in the initial condition to a mean of around four protocell types or 'quasi-species' at any one instant (figure 2b).

**Figure 3.**
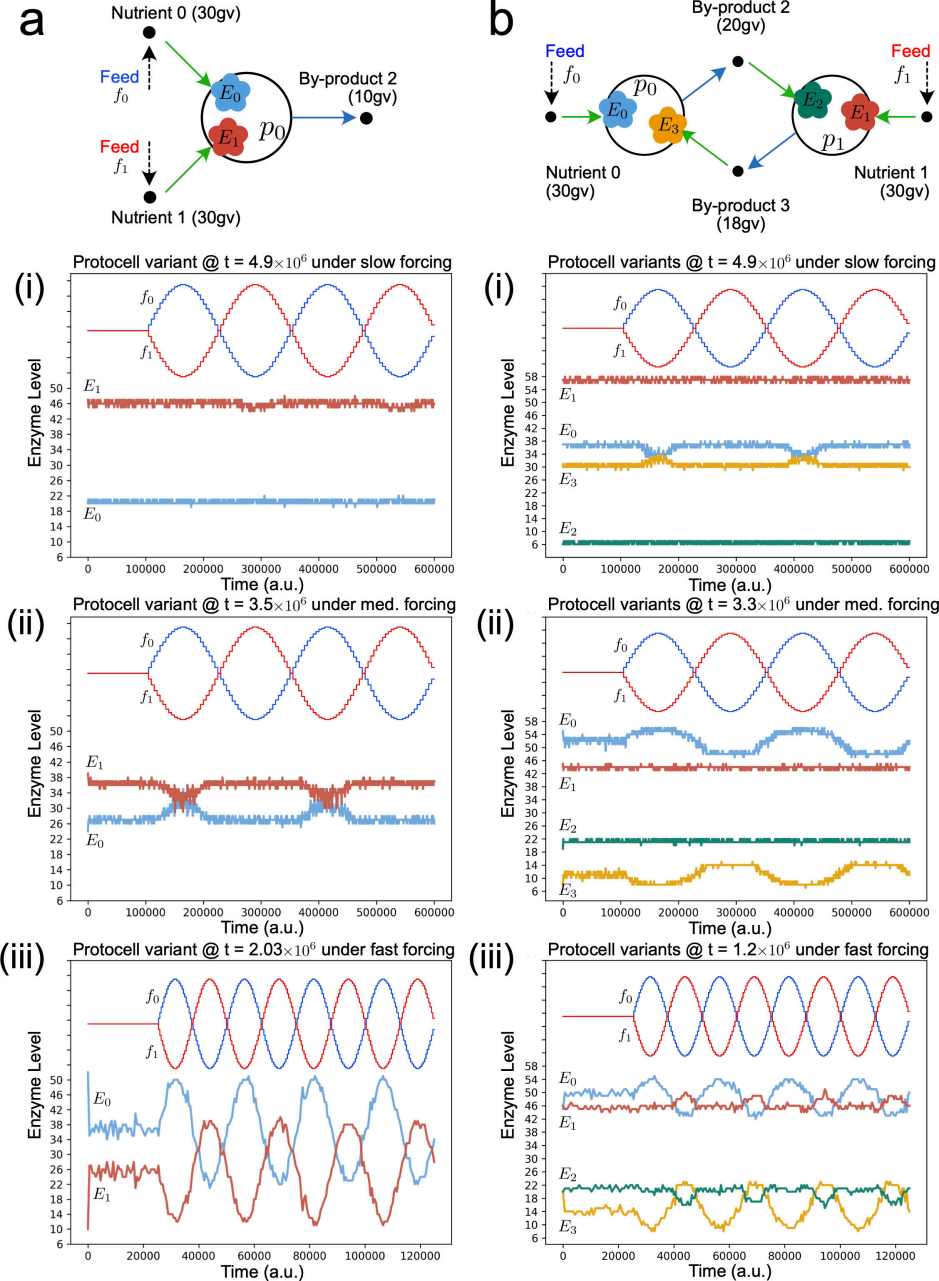
Dynamic responses of evolved regulatory networks in protocell types isolated from the main evolutionary lineage in simulations from figures 2 and 4. Protocell types are added to a new reactor in 100 copy number and exposed to nutrient cycling. Evolutionary mutations over time are disabled (no phylogenetic adaptation) and thus the source of enzyme level changes originates solely from regulatory networks, exposing what they have 'learned'. (a) For the single-protocell type simulations in figure 2. (b) For the two-protocell type ecology simulations in figure 4.

If nutrient abundance is varied as a slow sine wave with period *T* = 2.5 × 10^6^ time units, enzyme levels across the evolutionary lineage of protocell types now track the forcing, in both the −REG and +REG cases (figure 2d). In the −REG case, more of a lag occurred between the change in abundance of nutrient in the reactor feed and the subsequent change in internal enzyme level. This can be rationalized by noting that $p_{0}$ has a shorter phenotype size (of 4) in the −REG control condition, which leads to a decreased frequency of variants and hence a longer wait time for ‘phylogenetic’ adaptation (i.e. adaptation cropping up from evolutionary modifications). Conversely, $p_{0}$ in the +REG case has a greater internal complexity, reflected by a longer phenotype size (45), and this yields an increased frequency of variants. Additionally, after such a *functional* expansion, in the longer phenotype there exist many regulatory network parameters that can subtly affect enzyme levels. These latter two factors allow more synchronized adaptation to the varying nutrient source in the +REG case. It is important to remark that in the +REG case (under constant or slowly changing nutrient concentrations), the adaptation is still purely phylogenetic: the regulatory network is not functionally modifying the dynamics of the enzyme levels. This is evidenced in figure 3a(i), where a +REG protocell type at the end of the evolutionary lineage is tested against nutrient forcing, but with evolutionary variants disabled. In this case, the regulatory network does not dynamically respond to the nutrient forcing.

**Figure 4.**
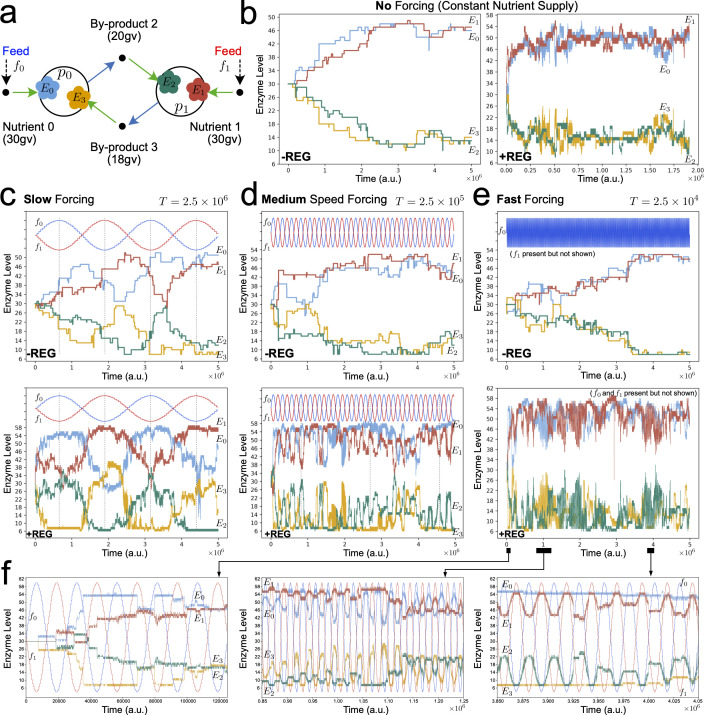
Dynamics of internal enzyme levels across the evolutionary lineage of a mutualistic ecology consisting of two protocell types, without (−REG) and with (+REG) potential to develop regulation mechanisms, under different nutrient forcing conditions. (a) Reactor starts with two protocell populations (*n* = 100 each) that cross-feed from each other. Enzyme dynamics across the evolutionary lineage under: (b) constant nutrient supply (f_0_ = f_1_ = 200 conc. units); (c) slow sinusoidal nutrient forcing where nutrients 0 and 1 oscillate between 40 and 200 conc. units, and are supplied 180° out of phase to the reactor; (d) medium-speed sinusoidal nutrient forcing; (e) fast sinusoidal nutrient forcing. (f) Zoom plots of enzyme dynamics in (e). Note that on all plots, nutrient forcing sine waves f_0_ and f_1_ use a separate *y*-axis that is not shown.

Under medium-speed forcing of nutrient abundance (sine wave period *T* = 2.5 × 10^5^ time units), effective phylogenetic adaptation is no longer possible in the −REG case (figure 2e, top). For the simulation parameters used, in the −REG condition, approximately 25 000 time steps are required to produce a significant variant (electronic supplementary material, table S5), giving only 10 significant variants per sine wave period. This is insufficient to properly adapt enzyme levels to the oscillation of nutrient abundance; hence enzyme levels evolve the null strategy as in the case of no forcing (figure 2c). By contrast, in the +REG case under medium-speed forcing, the behaviour is markedly different and there are frequent bursts of successful adaptation to the varying nutrients (figure 2e, bottom). For the simulation parameters used, in the +REG condition, approximately 5000 time steps are required to produce a significant variant (see electronic supplementary material, table S5), yielding an average of 50 variants per sine wave period. Adaptation to the nutrient source forcing in this case is a combination of both phylogenetic adaptation and partial emergence of functional regulatory networks. Figure 3a(ii) shows that a late-stage protocell type has evolved partially functional regulatory network enzyme dynamics when evolutionary variants are disabled. In this example, the regulatory network adapts internal enzymes *E*0 and *E*1 to follow external nutrient concentrations when [*c*_0_]>[*c*_1_], but not the other way around.

Finally, under fast nutrient forcing (sine wave period *T* = 2.5 × 10^4^ time units), the −REG condition is again unable to utilize phylogenetic adaptation, and enzyme levels follow the null strategy, as in figure 2e (top). However, in the +REG case (figure 2f, bottom), fast nutrient forcing causes the protocell type to evolve a functional regulatory network. As there are only five significant variants born per nutrient oscillation under fast forcing (on average), phylogenetic adaptation is limited. As evidenced in figure 3a(iii), evolution tinkers with a regulatory network able to autonomously change import enzyme levels, over short timescales, in phase with the reactor nutrient forcing. It is interesting to note that even when an efficient regulatory solution has evolved, sometimes, owing to the complexity of the selection process, this solution can be temporarily outcompeted by a protocell type with a non-functional regulatory network. Thus, the evolution of functional regulatory networks has a punctuated or bursting behaviour (consistent with a rugged fitness landscape). This is seen in both the medium- (figure 2e, bottom) and fast-speed (figure 2f, bottom) forcing cases.

### (c) Case study 2: ‘minimal mutualism’

We also investigated the evolutionary emergence of regulation mechanisms in a minimal mutualistic protocell ecology (figure 4a). The mutual ecology already has an intrinsic robustness or 'null strategy' to deal with changing nutrient concentrations: the symmetric cross-feeding relationships meant that, when nutrient 0 was abundant, $p_{1}$ could cross-feed from $p_{0}$ through metabolic by-product 2, and when nutrient 1 was abundant, $p_{0}$ could cross-feed from $p_{1}$ through metabolic by-product 3, instead. Our aim was to see if regulatory networks would evolve to implement a more efficient, dynamic response whereby *E*0 in $p_{0}$ and *E*2 in $p_{1}$ would increase in synchrony as nutrient 0 became abundant, while *E*1 in $p_{1}$ and *E*3 in $p_{0}$ would increase in synchrony as nutrient 1 became abundant. Under constant nutrient inputs (figure 4b), for both the −REG and +REG cases, the cross-feeding ecology evolves the strategy wherein $p_{0}$ mainly feeds from nutrient 0 (by up-regulating *E*0), and $p_{1}$ mainly feeds from nutrient 1 (by up-regulating *E*1). The +REG case arrives faster at this strategy because of the increased frequency with which variants are produced. During evolution, again several quasi-species develop around the original two protocell types.

When nutrients vary as two slow sine waves in anti-phase (figure 4c), internal enzyme levels in the protocell types across the evolutionary lineage oscillate in-phase with nutrient availability (+REG), or in-phase but lagged (−REG), similar to the case study 1. Figure 3b(i) shows that phylogenetic adaptation is mainly responsible for the enzyme level changes, with a regulatory response that is, in fact, not adequate (it actually brings *down E*0 levels when nutrient 0 is present in the medium). Increasing nutrient abundance to a medium oscillation speed (figure 4d) demonstrates that phylogenetic adaptation alone is insufficient to change enzyme levels in the −REG case, and enzyme levels over the evolutionary lineage become similar to the no-forcing case (figure 4b). In the +REG case, enzyme levels do track nutrient abundance, and this is caused by a combination of phylogenetic adaptation and short-term dynamics of the regulatory networks (figure 3b(ii)).

Using a fast oscillation speed for nutrient abundance in the +REG case (figure 4e, bottom) causes bursts of enzyme level activity in-phase with nutrient abundance. This activity is mainly controlled by regulatory networks (figure 3b(iii)) as phylogenetic adaptation is weak. Interesting to note is that under medium and fast forcing, $p_{0}$ and $p_{1}$ do not always change their enzyme levels in synchrony: there are times when $p_{0}$ is active alone, and other times when $p_{1}$ is active alone, and times when both are inactive. The ‘extreme regulation’ strategy (whereby *E*0 and *E*2 are at their maximum levels, while *E*1 and *E*3 are at their minimum, when nutrient 0 is abundant, and *vice versa* when nutrient 1 is abundant) does not evolve because nutrient uptake rates also depend on local chemical concentrations in the reactor, which diminish as uptake rates get higher.

### (d) Case study 3: ‘binary asymmetric ecology’

**Figure 5.**
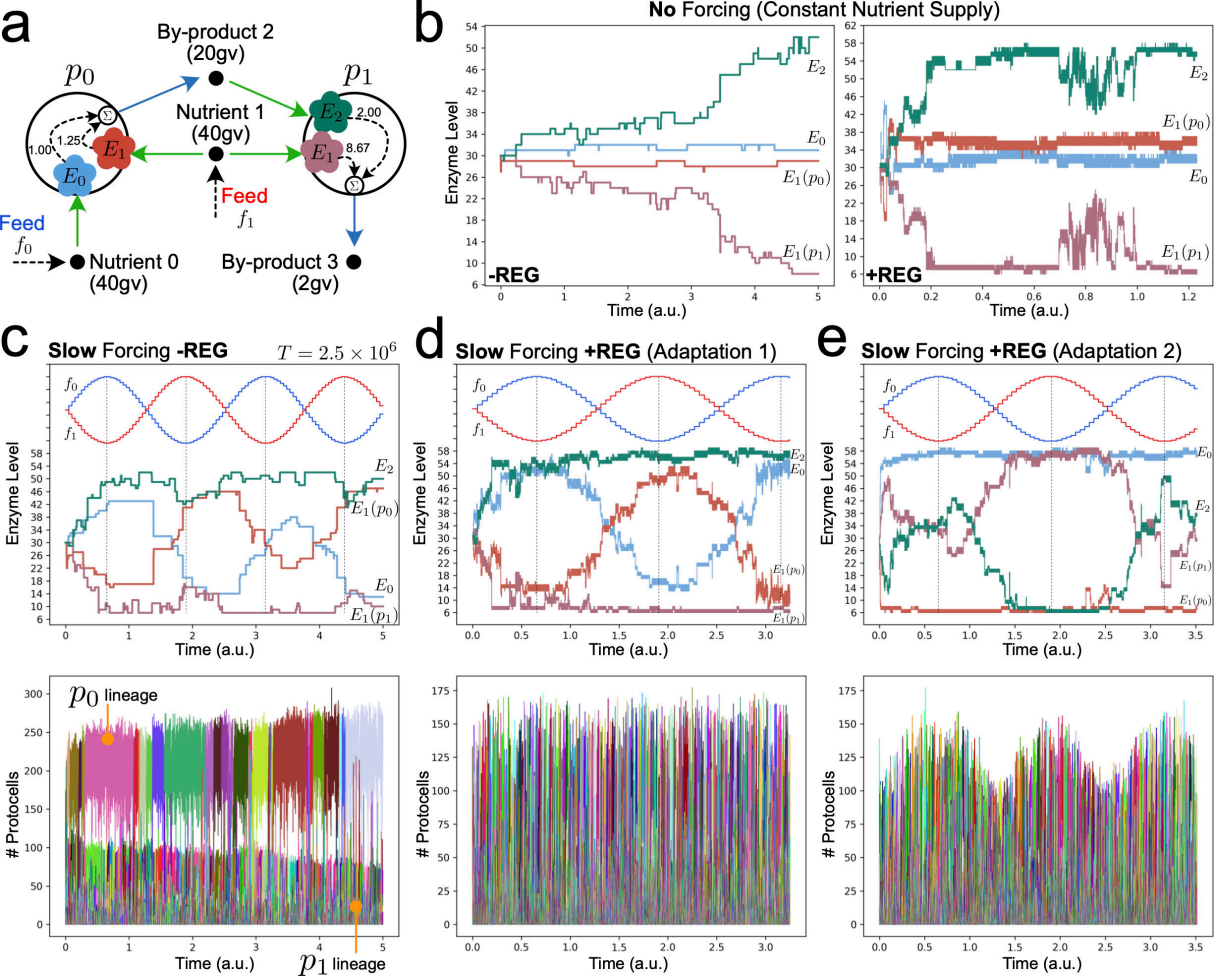
Different modes of phylogenetic adaptation of a co-operative/competitive minimal protocell ecology. (a) Reactor starts with two protocell populations (*n* = 100 each) that can cross-feed and/or compete for supplied nutrient 1. Long-term survival of this ecology under nutrient forcing and evolutionary mutations is highly sensitive to the initial conditions used (unlike ecologies in figures 2 and 4). Initial metabolic weights used for all simulations are shown. Enzyme dynamics across the evolutionary lineage under: (b) constant nutrient supply (f_0_ = f_1_ = 200 conc. units); (c–e) slow sinusoidal nutrient forcing. When protocells have the potential to develop regulatory mechanisms (+REG) under slow forcing conditions, then qualitatively different modes of phylogenetic adaptation can be achieved from the same initial condition (as shown in (d) and (e)). Both modes have distinct population dynamics. For the parameters used, functional regulatory networks did not develop in this ecology. Furthermore, the ecology could not survive medium-speed or fast forcing conditions.

As a final case, we investigated a protocell ecology that featured both cross-feeding and competition (figure 5a). With potential for competition, survival of this ecology was much more sensitive to initial conditions as compared with case studies 1 or 2. We found that, starting with particular metabolic weights (like those shown in figure 5a), this ecology could be sustained when evolving under no nutrient forcing (figure 5b) and under slow nutrient forcing (figure 5c–e), but not under medium-speed or fast nutrient oscillations. As such, regulatory networks with functional dynamics did not evolve in this scenario. However, the larger phenotypic space in the +REG case still allowed this ecology to exhibit another interesting effect. Starting from identical initial conditions, depending on stochastic fluctuations, protocells could show two qualitatively different modes of phylogenetic adaptation. Either the $p_{0}$ lineage adapted to the slow nutrient oscillations and the $p_{1}$ lineage always cross-fed from by-product 2 (figure 5d), or the $p_{0}$ lineage did not adapt and instead the $p_{1}$ lineage selectively cross-fed, adapting to the oscillating abundance of metabolic by-product 2. These different modes of adaptation were accompanied by distinctive population dynamics.

## 4. Discussion and outlook

These were our initial inroads into the question of how regulation could have started having significant impact on the evolutionary trajectories of growing and dividing protocell populations, within a framework that allowed cross-feeding relationships among them. For two of the three different minimal ecologies simulated, we found that protocells *do* evolve 'lac-operon-like' behaviours, switching their nutrient preferences spontaneously when the reactor is fed with fast-changing chemical precursor concentrations. For the homogeneous population case (case study 1) and the mutualistic ecology case (case study 2), the driving force behind the appearance of such a regulatory strategy is the effectiveness of dynamic metabolic adaptation to increase protocell growth rate when resources are limited and the environment is highly variable. However, the appearance of regulation cannot be generally assumed, given the non-trivial combination of—both endogenous and exogenous—factors involved, including high sensitivity to the initial ecological topology (as evidenced by case study 3). Besides, when regulation does arise, we found that it generally occurred in bursts, rather than being present continuously.

Hence, there is scope for much further work and reflection. Let us start with a note on the complexity of the dynamics involved in our simulations. Nothing should be taken for granted, really, as the last case study has shown us. One could have expected, from the results gathered in the first (‘single-protocell species’) and second (‘minimal mutualism’) scenarios, that regulatory behaviour was going to emerge also in the third (‘binary asymmetric ecology’), as the most adequate strategy to face environmental perturbations, especially when their frequency is increased. But this was not obvious. Of course, more simulation runs and initial conditions/parameters should be tried (including some comparative analysis with other theoretical approaches that could be addressing similar problems: e.g. [36–39]). Yet, the diverse, nonlinear combination of factors and scales within our computational approach makes predictability a real issue. Similarly, the degree of success of somatic/ontogenetic adaptive mechanisms (in comparison with evolutionary/phylogenetic ones—and also in conjunction with the relative frequency of external oscillations) is not easy to guess, despite the apparent general trend observed in the first two cases: i.e. the more stringent the environmental forcing, the more relevant regulatory behaviour seemed to turn. Still, in the third case, under rapidly varying conditions, regulation did not show up in time (provoking the actual collapse of one of the interacting protocell species).

Another point to be remarked is the fact that evolution and regulation are not disconnected from each other but, rather, asymmetrically coupled dimensions of the same phenomenon: namely, the transformation of the stochastic dynamic behaviour of protocell populations at longer-term and wider scales. Of course, this somehow derives from our theoretical premises and modelling assumptions, but it was confirmed by the simulation results obtained so far. Evolution (together with the presence of an external variation pattern ‘susceptible to assimilation’ by the protocells—irregular modifications of the boundary conditions would be much harder to deal with) is necessary for the emergence of regulation; in turn, the appearance of regulatory mechanisms modifies the evolutionary potential of the system (not just in somatic terms but also in a strictly phylogenetic sense—as was clear in the last case study, where the option for regulation, although a possibility, did not fructify). That is due to the increase in phenotypic space and, thereby, in the possibilities for mutation available to protocells. Thus, when regulatory mechanisms start operating in a system/population, the corresponding functional space is expanded and, with that, also evolvability *per se*. Indeed, the question is not just how big the number of state variables and parameters gets, but the amount of possible states that become accessible when all of them are put together.^4^ Effective regulation (i.e. the active use of the flexible correlation function added to the algorithm) plays a double role in this context: it expands *the space of the possible* and, in the same move, it provides new means to explore that space, enabling protocells to choose *actual* pathways/behaviours that happen to be better solutions to the challenges they face.

Nevertheless, the search for eventual stationary states, dynamic attractors or just optimized solutions where all the relevant factors (metabolic, ecological and evolutionary) are consistently integrated is not straight-forward, given the number of components, constraints and non-trivial, multi-scale causal loops we attempt to capture within the same modelling framework. When this project started, we considered making things even harder for the algorithm, but those initial ideas will have to wait for future implementations. For instance, the plastic ‘memory function’ has been tried, so far, without any additional production/maintenance cost, which is not realistic: phenotypic plasticity does not usually come for free (see [40]). In a prebiotic context, any material subsystem devoted to assimilation/detection and regulatory tasks should, indeed, take a significant part of the energy budget of a protocell (even if this is relatively smaller than the production/maintenance of other, more basic, functional components of the system). Strong simplifications were also made regarding the metabolic functioning of each protocell, whose real intricacies may have important evolutionary and ecological implications (see [34,41,42] or [43]). Likewise, spatial constraints do not play much of a role in our well stirred, flow-reactor setting, although we are perfectly aware of their relevance, not only at the level of the individual (which is something that we have worked on, previously, from different approximations [3,44]), but also in the collective and interactive domain (for instance, in the emergence of cross-feeding relationships [43]).

An obvious extension of the work reported here, which has been mainly focused on micro-evolutionary dynamics and minimalistic ecological cases, would involve a higher degree of ‘protocell species diversity’ (i.e. more heterogeneity in terms of metabolic ‘ways of life’, or ‘diets’, in the population) and, thus, also the development of more complicated protocell−protocell relationships. Araudia already presents the simulation options and features required for that research, so the potential is clearly there, but it has not been exploited yet. Even though we tried scenarios with various diets/species, these were kept constant in most of our simulation runs to date. Yet, the plan is to explore more complex ecological settings, either involving a bigger number of protocell species already in our initial conditions, or allowing their appearance during the simulation runs—to monitor, then, whether or not the corresponding ‘niches’ (new biomass pieces) remain interconnected and dynamically/structurally stable thereafter. Our strategy here has been to begin with simple (binary) cases, but the competitive exclusion principle may be operating more strongly in those scenarios (which would be—partly—the reason for the results obtained in our third case study). Indeed, there is evidence that multispecies microbial communities emerge and remain stable when a minimal threshold of diversity is crossed [45].

Along these lines, various kinds of changes in the way protocell diets have been implemented should be tried in future work: (i) making possible, in the course of our simulations, the modification in the nutrients and/or by-products of any given protocell metabolism in the population; (ii) exploring ‘co-consumption’ scenarios, in which secreted compounds are re-utilized as nutrients by the same species [46]—a possibility that was ruled out for our current simulation runs; (iii) transforming the ‘OR’ nutrition function (which we have used by default, up to now) into a more demanding ‘AND’ (which would apply to other specific situations—e.g. carbon- versus nitrogen-bearing substrate consumption [47]); (iv) letting other intermediary compounds play additional roles in protocell–protocell interactions (e.g. appearance of toxins); (v) including the option of secreting/detecting compounds to/in the environment that are not being used as nutrients/metabolites with a direct fitness cost (e.g. indirect ‘signals’ or chemical cues [48]). All this will surely open a whole new world of possibilities, where protocells unfold novel behaviours in terms of competition/cooperation strategies with their peers. Even if protocell capacities to switch or modulate their nutrient preferences, as were studied here, may already be taken as rudimentary forms of agency, it is in this wider context where the emergence of prebiotic *adaptive* agents could be more fruitfully addressed, because ‘speciation events’ (or ‘macroevolutionary transitions’) should transform protocell interactive features more deeply—making them, at the same time, subject to regulation.

In conclusion, we can say that the theoretical and computational modelling work presented in this article has provided interesting insights into the problem that we were initially addressing, especially by demonstrating that protocells can ‘learn’ from their interactions with the environment and perform actual regulatory behaviours that operate in somatic times, making them, in effect, more robust. Through that effort, we may have contributed to unveiling some aspects of the rather intricate link between regulation and evolution in the natural world, whereas the goal of connecting regulation with the prebiotic evolution of more sophisticated forms of agency, on the way towards proper biological agency, awaits future endeavours.

## Data Availability

Documentation on how to install and use the Araudia platform can be found at https://araudia.readthedocs.io/. Python code is open source and available under a GPL v3 license from repository https://bitbucket.org/ben_s_e/araudia. Results of all simulation runs for this study have been archived at [49]. Supplementary material is available online [50].

## Footnotes

^1^
The term ‘sub-species’ is the adequate one here, because we keep the ‘species’ category for a (larger) group of protocells that share a common ‘diet' or ‘ecological niche’ (i.e. the same set of nutrients/by-products in the chemostat, regardless of the values of all other simulation parameters). Although our computational platform allows ‘speciation events’ (i.e. the stochastic appearance of protocell variants that imply a change of diet), we are not reporting any simulation results that include such events yet. In other words, we are focusing, for the time being, on ‘micro-evolution’ dynamics. Nevertheless, we would like to keep this terminology, in view of future expansions of the current work towards the exploration of ‘macro-evolutionary’ transitions.

^2^
This also helps us circumvent the problem of proposing a more specific ‘recording mechanism’, which would have to be highly speculative, given the current state of affairs in the field of prebiotic evolution.

^3^
It is important to note that this implementation allows a broad and flexible control over enzyme levels to evolve: we do not require that dynamic metabolic adaptation necessarily maximizes the growth rate (as explored in some studies—e.g. [34]).

^4^
Our modelling approach can be interpreted as a ‘constructive dynamical system’, where state variables (protocell population levels, reactor chemical levels, enzyme levels) change over time under fixed parameters; yet, at longer temporal scales, evolution can also change parameters (metabolic network weights, regulatory network weights, etc.), as well as the actual number of state variables (e.g. when new protocell types arise). In this way, the evolutionary algorithm transforms the structure of the (stochastic) dynamical system itself, throughout the simulation.
